# Down syndrome iPSC model: endothelial perspective on tumor development

**DOI:** 10.18632/oncotarget.27712

**Published:** 2020-09-08

**Authors:** Mariana Perepitchka, Yekaterina Galat, Igor P. Beletsky, Philip M. Iannaccone, Vasiliy Galat

**Affiliations:** ^1^Department of Pediatrics, Northwestern University Feinberg School of Medicine, Chicago, IL, USA; ^2^Developmental Biology Program, Stanley Manne Children’s Research Institute, Ann & Robert H. Lurie Children’s Hospital, Chicago, IL, USA; ^3^Institute of Theoretical and Experimental Biophysics, Russian Academy of Sciences, Pushchino, Russia; ^4^Department of Pathology, Northwestern University Feinberg School of Medicine, Chicago, IL, USA; ^5^Robert H. Lurie Comprehensive Cancer Center, Northwestern University Feinberg School of Medicine, Chicago, IL, USA; ^6^ARTEC Biotech Inc, Chicago, IL, USA; ^*^Co-first author

**Keywords:** Down syndrome, iPSC-derived endothelial model, T21 genome-wide Implications, meta-analysis, tumor microenvironment

## Abstract

Trisomy 21 (T21), known as Down syndrome (DS), is a widely studied chromosomal abnormality. Previous studies have shown that DS individuals have a unique cancer profile. While exhibiting low solid tumor prevalence, DS patients are at risk for hematologic cancers, such as acute megakaryocytic leukemia and acute lymphoblastic leukemia. We speculated that endothelial cells are active players in this clinical background. To this end, we hypothesized that impaired DS endothelial development and functionality, impacted by genome-wide T21 alterations, potentially results in a suboptimal endothelial microenvironment with the capability to prevent solid tumor growth.

To test this hypothesis, we assessed molecular and phenotypic differences of endothelial cells differentiated from Down syndrome and euploid iPS cells. Microarray, RNA-Seq, and bioinformatic analyses revealed that most significantly expressed genes belong to angiogenic, cytoskeletal rearrangement, extracellular matrix remodeling, and inflammatory pathways. Interestingly, the majority of these genes are not located on Chromosome 21. To substantiate these findings, we carried out functional assays. The obtained phenotypic results correlated with the molecular data and showed that Down syndrome endothelial cells exhibit decreased proliferation, reduced migration, and a weak TNF-α inflammatory response. Based on this data, we provide a set of genes potentially associated with Down syndrome’s elevated leukemic incidence and its unfavorable solid tumor microenvironment—highlighting the potential use of these genes as therapeutic targets in translational cancer research.

## INTRODUCTION

Down syndrome (DS) is commonly evaluated on the basis of physical and clinical traits resulting from genomic alterations caused by a trisomy of Chromosome 21 (T21) [1]. DS occurs at a frequency of 1/700–1/800 births, and the frequency increases with maternal age [2]. Initially, the dominant perspective was that DS phenotypes resulted from extra gene dosage effects solely relative to T21. Furthermore, even though Chromosome 21 is considered to host approximately 350 genes, research efforts were directed toward a small subset of genes clustered around the DS critical region (DSCR) [3]. More recent studies, however, suggest that there are potentially many causative genes in DS distributed over larger regions of Chromosome 21 [4], and such gene dysregulation may impact up to one-third of disomic genes [5]. Additionally, transcriptome and protein analyses have shown that this Chromosome 21 dosage effect can induce gene overexpression and/or underexpression on a genome-wide level [6]. Such genome-wide expression dysregulation in DS was actually noted in a study on fetal fibroblasts of monozygotic twins discordant for T21 [7].

Studies utilizing fetal tissue and animal models have provided valuable input into understanding DS clinical and physical features. This being said, studies at the fetal stages are limited by ethical and technical considerations, and mouse models do not fully recapitulate human DS developmental traits [8–10]. Pluripotent stem cell models are a powerful alternative that can be employed to further our understanding of the molecular and biochemical effects of T21 on human development [11]. Our previous research, amongst others’, has shown that induced pluripotent stem cells (iPSCs) progress through major embryonic developmental stages [12–14], and by utilizing DS iPSCs, this opens a direct line into investigating DS phenotypic traits and genotypic implications.

From a genotypic standpoint, previous studies focusing on DSCR1 and DYRK1A genes, located on the extra copy of Chromosome 21, showed that DS individuals have a unique cancer profile. On one hand, DS children have a 500-fold risk of developing acute megakaryoblastic leukemia (AMKL) and a 20-fold risk of being diagnosed with acute lymphoblastic leukemia (ALL) [15–17]. On the other hand, the DS genotypic profile is also associated with reduced solid tumor growth [18, 19]. Such an unusual cancer profile may potentially exemplify a dynamic interplay of genetic mutations and the construction of a tissue-specific microenvironment that hinders the expansion of the pre-metastatic solid tumor niche.

Within this suboptimal microenvironment, the tumor would be unable to employ strategies that involve the use of and communication with host cellular machinery. Examples include: secretion of extracellular vesicles, enhancing transcription of extracellular matrix (ECM) and cytoskeletal remodeling enzymes, induction of angiogenesis, vessel co-option, up-regulation of cytokine receptors, and recruitment of pro-inflammatory signaling molecules [20–23]. Endothelial cells, which have incredible plasticity/structural heterogeneity, support hematopoietic stem cell maintenance, and promote hematopoietic and immune processes, are highly employed by the solid tumor niche. To this end, we hypothesized that impaired DS endothelial development and functionality, impacted by genome-wide T21 alterations, potentially results in a suboptimal endothelial microenvironment with the capability to prevent solid tumor growth.

This work is the first study of DS iPSC-produced endothelial cells (iECs). Our group, as well as other researchers, has developed robust technologies of endothelial cell derivation from iPSCs [24–28]. To test our hypothesis, we employed such technology to assess the molecular and phenotypic differences of endothelial cells differentiated from DS and euploid iPSCs. Our microarray, RNA-Seq, and bioinformatic analyses revealed that most of the differentially expressed genes belong to proliferative (angiogenic), cytoskeletal rearrangement, ECM remodeling, and inflammatory pathways. All of these pathways incorporate crucial biochemical mechanisms that the solid tumor niche potentially alters for metastatic initiation and progression. Interestingly, the majority of the significantly expressed genes within these pathways were not located on Chromosome 21. These findings, confirmed by functional assays, may prove to be useful in ongoing DS clinical research and provide a new perspective on tumor development, which can aid future cancer-related studies.

## RESULTS

### Characterization and bioinformatic functional assessment of iPSCs and iECs

To begin evaluating the DS endothelial genotype and phenotype, cellular characterization experiments were initially performed to verify stem cell pluripotency prior to endothelial differentiation. Confirmation of endothelial lineage commitment followed afterward. For these experiments, the following cell lines were employed: patient-derived skin fibroblasts (FB1, FB2), a commercially purchased embryonic stem cell line (H9-ESC), induced pluripotent stem cells (SR2-iPSCs DSV-iPSCs, isoDSV-iPSCs), and endothelial cells (commercially purchased HUVECs, DSV-iECs, isoDSV-iECs, SR2-iECs, H9-iECs). The SR2-iPSCs and DSV-iPSCs were established *via* over-expression of Sox2, c-Myc, Oct4, and Nanog in commercially purchased fibroblasts. Additional differentiation and characterization information is provided in [13, 29, 30]. Regarding isoDSV-iPSCs, this cell line was established as a result of the spontaneous loss of an extra copy of Chromosome 21 in DSV-iPSCs. These three iPS cell lines were differentiated into endothelial cells (SR2-iECs, DSV-iECs, isoDSV-iECs), which were previously characterized in [24, 31, 32].

**Figure 1:**
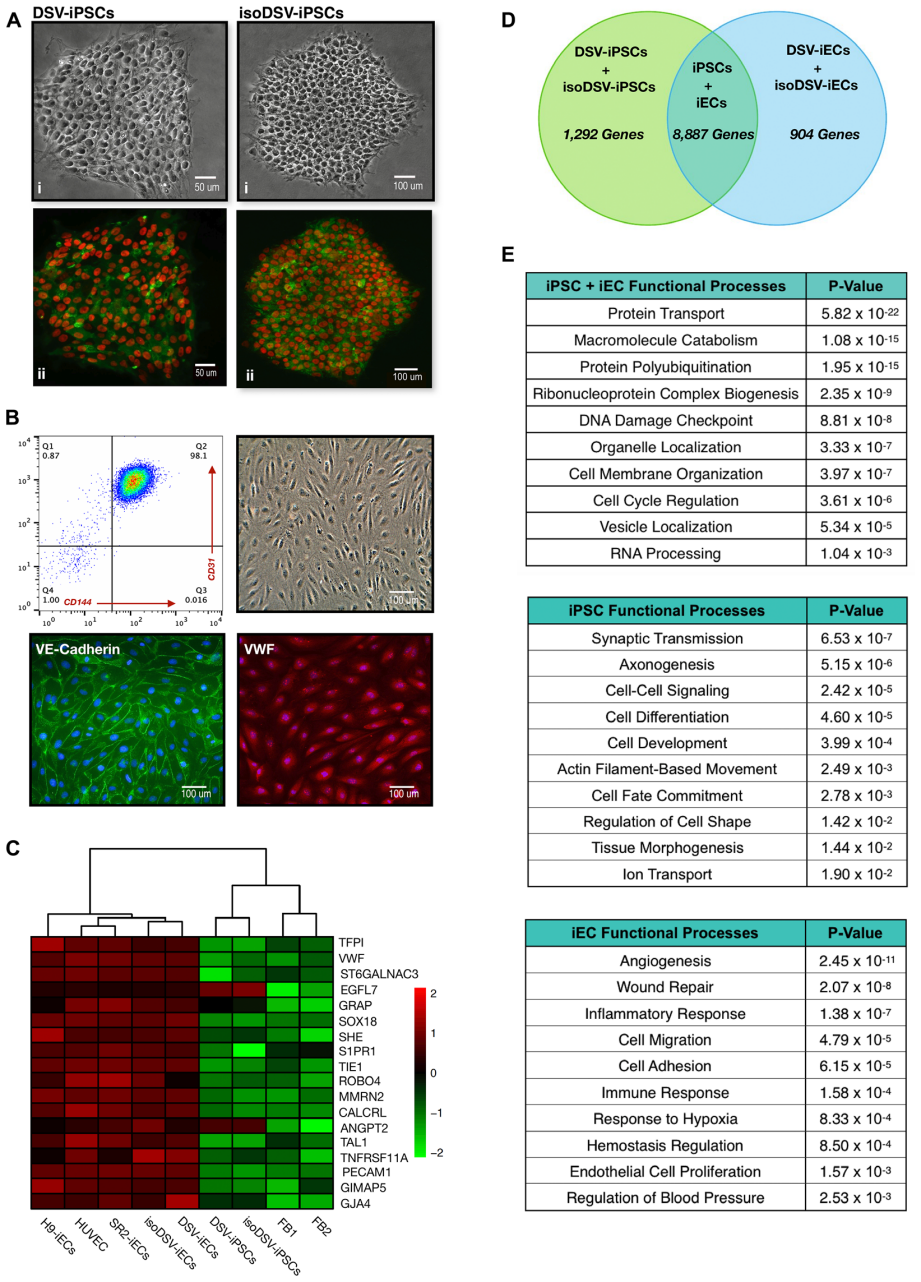
Characterization of disomic and trisomic iPSCs and iECs. (**A**) (i) Phase contrast microscopy images of the trisomic and isogenic pair: DSV-iPSCs and isoDSV-iPSCs. (ii) Immunocytochemistry images showing TRA-1-60 [green] and OCT4 [red] co-expression in DSV-iPSCs and isoDSV-iPSCs. (**B**) Representative images: (1) flow cytometric analysis of iECs demonstrating homogeneity of CD31 and CD144 co-expression; (2) Cobblestone endothelial morphology; (3) Immunocytochemistry showing positive expression of cell-surface marker VE-Cadherin (CD144); (4) Immunocytochemistry data confirming positive expression of von Willebrand factor (VWF). Cell nuclei were stained with DAPI [blue]. (**C**) Heatmap showing vascular-related gene expression correlation and cell line clustering amongst HUVEC, disomic SR2-iECs, isoDSV-iECs, DSV-iECs, isoDSV-iPSCs, and DSV-iPSCs. FB1 and FB2 cell lines are fibroblasts. The microarray expression data has been log-transformed. (**D**) Venn diagram generated *via* Network Analyst showing the number of microarray genes unique to and shared between DSV-iPSC, isoDSV-iPSC, DSV-iEC, and isoDSV-iEC cell lines. (**E**) Statistically significant functional processes that incorporate the genes in the venn diagram. Processes were obtained *via* Network Analyst’s enrichment analysis (GO:BP database).

For pluripotency verification, immunocytochemistry was carried out to ensure pluripotency marker expression in H9-ESCs, SR2-iPSCs, DSV-iPSCs, and isoDSV-iPSCs. Co-expression of TRA-1-80/Nanog and TRA-1-60/Oct4 was evident in all cell lines. TRA-1-60/Oct4 expression for DSV-iPSCs and isoDSV-iPSCs is provided (Figure 1A). Following endothelial differentiation, FACS analysis was performed to confirm endothelial lineage-specific marker expression. The acquired endothelial cells (H9-iECs, DSV-iECs, isoDSV-iECs, SR2-iECs) were CD34^+^/CD31^+^(PECAM-1)/CD144^+^(VE-Cadherin). The cell lines also displayed the characteristic cobblestone morphology, and an additional immunocytochemical evaluation confirmed expression of von Willebrand factor (VWF) and VE-Cadherin (Figure 1B). To further ensure lineage commitment, a heatmap of characteristic endothelial genes was generated from microarray data *via* R Studio. The results show a clear delineation between endothelial, iPSC, and fibroblast gene expression levels. Additionally, by employing a clustering algorithm, the heatmap provides another layer of distinction with regard to disomy vs. trisomy. The disomic endothelial cells (SR2-iECs, HUVECs, H9-iECs), trisomic and isogenic endothelial pair (DSV-iECs, isoDSV-iECs), trisomic and isogenic induced pluripotent stem cell pair (DSV-iPSCs, isoDSV-iPSCs), and fibroblasts (FB1, FB2) have all been appropriately paired (Figure 1C).

To complement these results, a bioinformatic functional analysis was performed utilizing the Network Analyst, a comprehensive gene analysis platform [33]. The microarray dataset utilized in this analysis incorporated DSV-iPSC, isoDSV-iPSC, DSV-iEC, and isoDSV-iEC cell lines. First, we obtained an overview of the number of iPSC-specific genes, iEC-specific genes, and shared genes within the dataset (Figure 1D). We then performed a functional enrichment analysis on each gene group. The reference database was Gene Ontology: Biological Pathways (GO:BP). The iPSC + iEC functional processes reflect general cell regulatory mechanisms critical to cell survival, which explains the large gene number (8,887) shared amongst the cell lines. The other data tables include functional processes that specifically correlate with iPSC and iEC lineages, respectively. All reported functional processes are statistically significant (Figure 1E).

### Comparative bioinformatic cancer gene analyses of DSV-iECs

Following characterization, we performed comparative gene analyses to study the potential genomic impact of T21 with respect to aberrant DS endothelial development, increased leukemic prevalence, and decreased solid tumor incidence. We compared clinical oncology RNA-Seq data (oncogene/tumor suppressor perspective) and RNA-Seq data from DSV-iEC and isoDSV-iEC cell lines. In order to generate the clinical oncology gene expression dataset, we utilized The Cancer Genome Atlas (TCGA) [34, 35] and the OncoKb database [36]. To complement our DS endothelial RNA-Seq expression data, we referenced the Cancer GeneticsWeb, which integrates data from several databases such as OMIM, PubMed, GO, GeneCards, and others [37]. Additionally, to further ensure an accurate cancer type-to-gene correlation for both datasets, the OncoLnc database was used as another resource. OncoLnc couples clinical oncology data (21 cancer cases) with mRNA expression levels [38].

#### Chromosome 21 cancer-related genes

**Figure 2:**
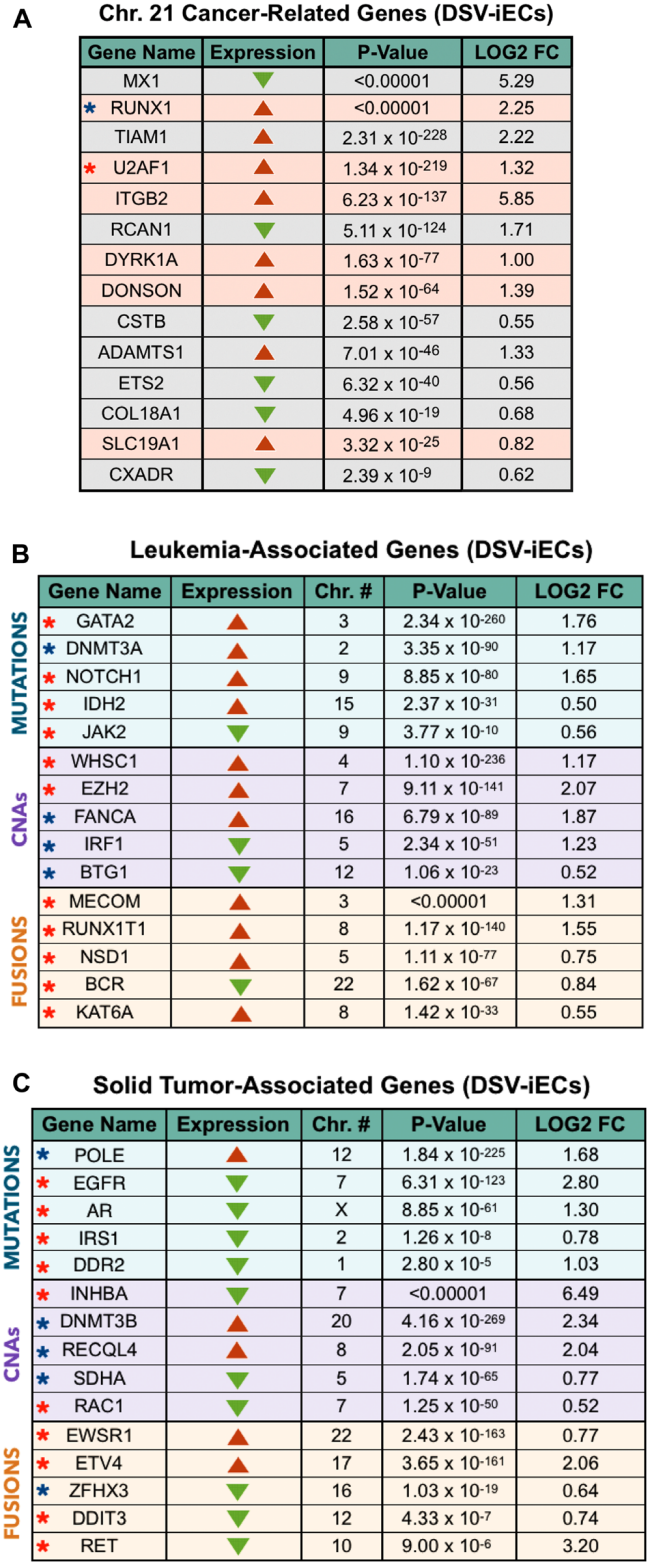
DS endothelial perspective on tumor development: chromosome 21 cancer-related genes, leukemia-associated genes, solid tumor-associated genes. (**A**) Significantly expressed leukemia-associated [red] and solid tumor-associated [grey] genes mapped along Chromosome 21. The blue star represents a tumor suppressor, and the red star highlights an oncogene. Red arrows symbolize up-regulation, and green arrows are representative of down-regulation. (**B** and **C**) Top 5 significantly expressed, genome-wide leukemia and solid tumor-associated oncogenes [red stars] and tumor suppressors [blue stars] per most frequent alteration type (mutations, CNAs, fusions). Red arrows show up-regulated expression, and green arrows represent down-regulated expression. The *p*-value, fold change, and expression data for all gene panels was obtained from RNA-Seq analysis. Additionally, every gene panel shows DSV-iEC expression values compared to isogenic control (isoDSV-iECs).

Throughout the years, a significant number of cancer-related genes, mapped along Chromosome 21, have been identified. We evaluated our DSV-iEC model with respect to the gene expression levels of 26 commonly studied, cancer-related genes on Chromosome 21 [37]. Genes with non-significant *p*-values and fold changes (FCs) < 0.5 were excluded from the analysis. The resulting gene list consisted of 14 differentially expressed genes (Figure 2A). We then utilized the OncoLnc database to assess which of these genes are predominately expressed in leukemias or solid tumors. The RUNX1, U2AF1, ITGB2, DYRK1A, DONSON, and SLC19A1 gene expression levels were highly elevated in AML cases. Our RNA-Seq data correlates with this clinical data by showing significantly up-regulated expression levels for all six genes in DSV-iECs vs isoDSV-iECs. The remaining 8 genes had elevated mRNA expression levels across several solid tumor cases, according to OncoLnc. In comparison to clinical data, 6 out of the 8 genes (MX1, RCAN1, CSTB, ETS2, COL18A1, CXADR) were significantly down-regulated in DSV-iECs whereas TIAM1 and ADAMTS1 genes were significantly up-regulated.

#### Leukemia and solid tumor–associated gene panels

To further investigate the genetic implications of T21 relative to tumor formation, we also assessed genome-wide differential expression between trisomic DSV-iECs and disomic isoDSV-iECs. Our approach was two-fold. First, we utilized TCGA to compile a list of 500 genes that are frequently altered in leukemias and 500 genes frequently altered in solid tumors. The OncoLnc database supplemented this portion of the analysis by providing additional confirmation as to which genes have a strong correlation with either leukemic or solid tumor types. We then organized the gene lists based on alteration category: mutations, copy number alterations (CNAs), and fusions. Afterward, we employed the OncoKb database to ensure that our gene lists contain significantly expressed and frequently altered oncogenes and tumor suppressors associated with at least 3 leukemic or 3 solid tumor types. Genes with non-significant *p*-values, FC < 0.5, and genes displaying a relatively equal contribution toward leukemic and solid tumor development were omitted from the analysis. Following this, we selected the top 5 significantly expressed genes per alteration category.

The leukemia-associated gene panel contains 11 oncogenes and 4 tumor suppressors (Figure 2B). Out of the 11 oncogenes, compared to isoDSV-iECs, DSV-iECs exhibited up-regulated expression of 9 oncogenes (mutations: GATA2, NOTCH1, IDH2; CNAs: WHSC1, EZH2; fusions: MECOM, RUNX1T1, NSD1, KAT6A). JAK2 (mutations) and BCR (fusions) oncogenes were down-regulated. With regard to tumor suppressors, DSV–iECs showed an equal divide: 2 up-regulated (mutations: DNMT3A; CNAs: FANCA) and 2 down-regulated (CNAs: IRF1, BTG1) genes. Overall, it seems that DSV-iECs are showing an up-regulated trend with regard to leukemia-associated oncogenes.

With regard to the solid tumor-associated gene panel, there are 10 oncogenes and 5 tumor suppressors (Figure 2C). 8 of the oncogenes have a down-regulated expression (mutations: EGFR, AR, IRS1, DDR2; CNAs: INHBA, RAC1; fusions: DDIT3, RET). EWSR1 and ETV4 (fusions) exhibit up-regulated expression. For the tumor suppressors, there are 3 up-regulated genes (mutations: POLE; CNAs: DNMT3B, RECQL4) and 2 down-regulated genes (CNAs: SDHA; fusions: ZFHX3). In comparison to the leukemia-associated gene panel, DSV-iECs potentially exhibit a greater inclination toward down-regulating solid tumor-associated oncogenes.

### Endothelial microenvironment: MetaCore pathway analysis of DSV-iECs

Observing the variability in oncogene and tumor suppressor expression levels in DSV-iECs vs isoDSV-iECs, we decided to perform bioinformatic pathway analysis utilizing the Clarivate Analytics MetaCore program. Our pathways of interest involved endothelial processes that solid tumors may exploit for growth and metastasis:

#### Proliferation pathways

According to MetaCore’s enrichment analysis, the pathway with the lowest *p*-value (1.987 × 10^-23^) involved Hypoxia Inducible Factor-1 (HIF-1) signaling (Supplementary Figure 1A). This significant *p*-value reflects the summation of expression values across HIF-1 pathway’s multiple gene targets. HIF-1 is a critical regulator of a wide array of physiological processes, including: angiogenesis, ECM remodeling, cytoskeletal rearrangements, and inflammation [39–42]. With regard to our dataset, the HIF-1 complex impacts downstream Endothelin-1 (EDN1) gene expression (*p*-value = 0, FC = 3.86), (Supplementary Figure 1B). Down-regulated in DSV-iECs, EDN1 is an angiogenic factor involved in cell proliferation, and its overexpression has been linked to tumor growth and metastasis [43, 44]. Further investigation of EDN1 signaling (EDN1/EDNRB) showed that this pathway can induce downstream ERK1/2 signaling, which is also down-regulated in DSV-iECs (Supplementary Figure 2A).

Since ERK1/2 is involved in cell proliferation, migration, and survival [45], we investigated its up and downstream targets in connection with the EDN1/EDNRB pathway. By referencing MetaCore’s published pathways and utilizing the Pathway Map Creator application, we identified the Integrin beta-3 (ITGB3) gene. Integrin beta-3 is activated by EDNR1/EDNRB and CCL2 pathways with ERK1/2 at the crossroads (Supplementary Figure 4 and Supplementary Figure 5A). Based on *p*-value (3.93 × 10^-275^), FC (6.28), and its capability to increase cellular survival and migratory potential, we selected ITGB3 as another gene of interest. The ITGB3 gene, like EDN1, is also down-regulated in DSV-iECs. Additionally, ITGB3 expression is increased during oxidative stress, which further implicates its involvement in the tumor microenvironment (TME) [46].

Further analysis of ERK1/2 upstream and downstream targets brought the Tissue factor (F3) gene (*p*-value = 4.66 × 10^-53^, FC = 4.31) to our attention. An upstream regulator of the ERK1/2 complex, the F3 gene is down-regulated in DSV-iECs (Supplementary Figure 2B and Supplementary Figure 4). F3 signaling activates a variety of molecular pathways through G-protein-coupled receptors (ex., EGFR pathway) and contributes to cellular proliferation. Overexpression of the F3 gene has also been associated with cell migration/cytoskeletal reorganization and tumor progression [47, 48]. As a result, F3 became an additional gene of interest linked to tumor development due to its proliferative and migratory impact.

#### Migration/cytoskeletal rearrangement and ECM remodeling pathways

In the TME, expression of proliferative factors is complemented by the initiation of the metastatic cascade: cancer cell invasion, intravasation, and extravasation. This motile phenotype stems from increased ECM elasticity, permeability, and degradation [49, 50]. We explored ECM remodeling pathways, and within the CCL25/CCR9 signaling pathway, we identified three downstream genes that showed the most extensive changes in their differential expression: MMP-1 (Matrix Metallopeptidase-1, *p*-value = 0, FC = 6.45), MMP–10 (Matrix Metallopeptidase-10, *p*-value = 1.97 × 10^-126^, FC = 6.13), and HAPLN1 (*p*-value = 0, FC = 6.99). MMP-1 and MMP-10 are down-regulated in DSV-iECs, while HAPLN1 is up-regulated. Furthermore, all three genes are involved in ECM composition and fluidity (Supplementary Figure 3A).

A discontinuous and unstable ECM composition triggers actin cytoskeletal rearrangements, which initiate changes in cell shape and promote migration. Analysis of cytoskeletal remodeling pathways revealed that DSV-iECs displayed a consistent down-regulation in the expression levels of major downstream cytoskeletal complexes, such as F-actin cytoskeleton and actomyosin (Supplementary Figure 3B). When evaluating the expression levels of the genes involved in the formation of these complexes, the ACTG2 gene had the lowest *p*-value (5.67 × 10^-31^) and a FC = 5.05. Additionally, the ACTG2 gene, as part of the downstream actin complex, also plays a key role in the TGF-β pathway, which is a major inducer of cell migration [51], (Supplementary Figure 3B).

#### Inflammation pathways

Changes in proliferative capacity and ECM composition or fluidity can stimulate expression of inflammatory response agents within a multitude of pathways that intertwine *via* the transcription factor NF-kB [52, 53]. As a result, we approached our inflammatory signaling analysis by focusing on significantly expressed up and downstream genes of pathways that incorporate NF-kB, which is down-regulated in our DSV-iEC model. More specifically, we focused on the crosstalk between NF-kB and the CCL2, IL-33, IL-1, and EGFR signaling pathways (Supplementary Figures 4–6). Our approach was two-fold: (1) analyze gene expression relative to each individual pathway; (2) study genes impacted by the interconnectivity of these pathways. In the first part of this analysis, the following upstream genes were the most significantly expressed: CCL2 (*p*-value = 0, FC = 6.93), IL-33 (*p*-value = 2.39 × 10^-238^, FC = 10.48), and IL-1β (*p*-value = 1.29 × 10^-61^, FC = 4.70), (Supplementary Figure 5 and Supplementary Figure 6A). Regarding downstream signaling, the APOE gene (*p*-value = 2.40 × 10^-88^, FC = 3.72, IL-1 pathway) and SERPINB2 (PAI2) gene (*p*-value = 1.55 × 10^-71^, FC = 6.88, EGFR pathway) showed significant differential expression (Supplementary Figure 6).

For the second part of the inflammatory analysis, the interconnectivity of the EGFR, IL-1, CCL2, and IL-33 pathways highlighted additional genes that are simultaneously regulated by several of these pathways. The extensive regulation of these genes correlates with significant differential expression. More specifically, the IL-6 gene (*p*-value = 9.12 × 10^-32^, FC = 6.97) was impacted by CCL2, IL-1, and IL-33 pathways; the CXCL1 gene (*p*-value = 0, FC = 5.71) was part of the IL-1 and IL-33 pathways; the IL-8 gene (*p*-value = 3.74 × 10^-149^, FC = 10. 52) was present in the IL-1, IL-33, and EGFR pathways (Supplementary Figures 5 and 6). As an added factor for consideration, irregularities in the expression levels of these upstream and downstream genes have been implicated in tumorigenesis [54–58].

#### Visualization and expression assessment of select pathway genes

**Figure 3:**
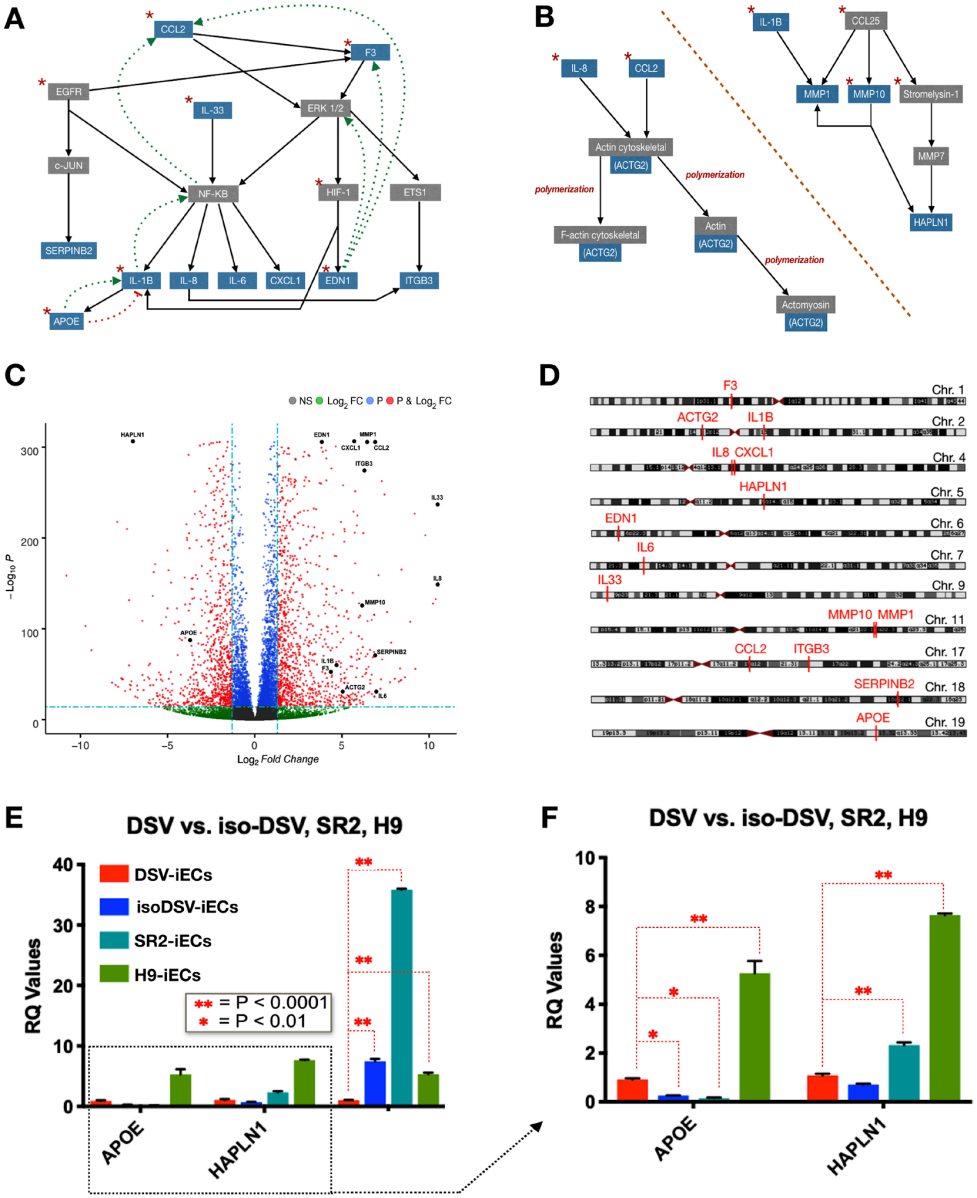
Bioinformatic pathway analysis and gene verification. (**A** and **B**) Schematic representations of proliferation + inflammation pathways [left] and cytoskeletal rearrangement + ECM remodeling pathways [right]. Genes of interest are highlighted in blue. Red stars symbolize pathway starting points. The dotted lines refer to activation [green arrows] and inhibition [red bar-headed lines] mechanisms. (**C**) Volcano plot showing the statistical significance of the selected genes of interest. In relation to DSV-iECs, the down-regulated genes are toward the right, and up-regulated genes are on the left. (**D**) Chromosome plot showing locations of selected genes. Chromosome locations were obtained *via* RNA-Seq analysis and the UCSC Genome Browser. (**E**) rtPCR verification of both microarray and RNA-Seq data with regard to APOE, HAPLN1, and CCL2 expression. (**F**) rtPCR data table with CCL2 removed to improve visualization of APOE and HAPLN1 expression. The rtPCR tables include data, presented as mean ± SEM, from three experimental replicates.

As a summation of the pathway analysis, schematic representations of the proliferation, migration, and inflammation pathways were created. The schematics show pathway interconnectivity and highlight the genes of interest (Figure 3A and 3B). Following pathway analysis, to better visualize the extent of differential expression, we constructed a volcano plot showing the top 7,000 genes within our RNA-Seq dataset. All of the 15 selected genes are significant with respect to *p*-value and FC (Figure 3C). We also mapped the chromosomal locations of all 15 genes. Interestingly, with regard to proliferation, cytoskeletal rearrangement, ECM remodeling, and inflammation pathways, these most significantly expressed genes were not located on Chromosome 21 (Figure 3D).

This observation paved the way for another assessment: evaluating how extensively endothelial lineage effects the expression levels of the select 15 genes following iPSC differentiation. Utilizing microarray data, we compared gene expression levels between DSV-iECs/DSV-iPSCs and isoDSV-iECs/isoDSV-iPSCs. DSV-iECs showed an up-regulation in all genes, except ITGB3, ACTG2, IL-1β, IL-6, APOE, and SERPINB2 (PAI2). isoDSV-iECs showed an up-regulation in all genes, except HAPLN1 and APOE. This result indicates that endothelial maturation promotes an up-regulatory expression trend with regard to the select 15 genes. This being said, the fact that DSV-iECs exhibited fewer up-regulated genes in comparison to isoDSV-iECs is worth to consider relative to T21 implications on endothelial development—potentially leading to a suboptimal endothelial microenvironment.

#### rtPCR verification

Out of the 15 genes, we performed rtPCR verification on CCL2, HAPLN1, and APOE genes. This selection stems from our focus on gene expression differences between iPSCs *vs.* iECs, up/downstream gene targets, and the interconnectivity of pathway interactions. With respect to our gene selection, CCL2 is a versatile upstream gene, and its pathway impacts proliferative, inflammatory, and cytoskeletal rearrangement mechanisms. CCL2 also effects the expression levels of important gene targets involved in tumorigenesis: VEGF, TNF-α, INF-gamma, HIF1-A, etc. Furthermore, CCL2 expression is down-regulated in DSV-iECs. HAPLN1 impacts ECM composition *via* proteoglycan affiliation. Its downstream signaling effects migratory cytoskeletal rearrangements and ECM remodeling. HAPLN1 is up-regulated in DSV-iECs. APOE is a downstream gene at the intersection of the IL-1B and TNF-α pathways. These pathways are both implicated in inflammatory regulation. APOE is up-regulated in DSV-iECs. rtPCR results confirmed the expression levels for all three genes (Figure 3E and 3F).

### iEC functionality

#### iEC development

**Figure 4:**
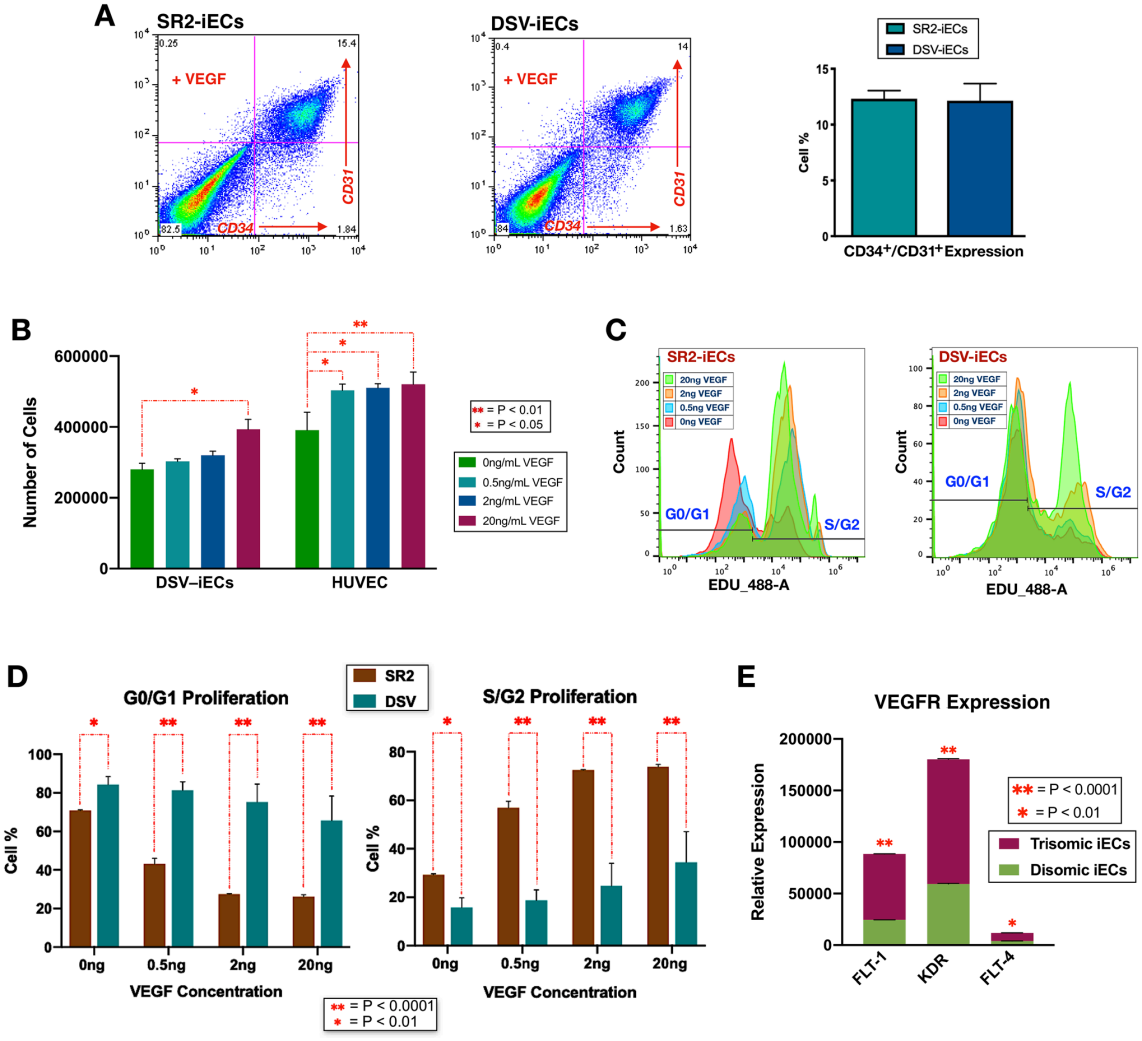
Endothelial differentiation efficiency, VEGF response sensitivity, and proliferative potential of disomic and trisomic iECs. (**A**) Flow cytometry results of DSV-iEC and SR2-iEC differentiation in the presence of VEGF. The endothelial differentiation efficiency table incorporates expression data from three replicates. (**B**) DSV-iEC and HUVEC cell counts following 0.5 ng/mL, 2 ng/mL, and 20 ng/mL VEGF addition. The cell counts incorporated three replicates. (**C**) Flow cytometry data from the EdU Proliferation Assay. For the SR2-iEC cell line, the data shows the G0/G1 cell percentage consistently decreasing as more cells are transitioning into the S/G2 cell cycles. DSV-iECs, despite showing a growing cell percentage in the S/G2 cell cycle phases, have a significantly larger percentage of cells in the G0/G1 phases. (**D**) All EdU flow cytometry data compiled into a histogram. Four replicates were included in the assay. (**E**) RNA-Seq data showing up-regulated expression of three VEGF receptors in DSV-iECs. All statistical data in the figure is presented as mean ± SEM.

Based on pathway and bioinformatic analyses, our gene expression data indicated that DSV-iECs have a genome-wide expression dysregulation in comparison to disomic control iECs. We were interested whether these genetic differences effected the formation of endothelium at the earliest stages of development. To gain more insight, we compared endothelial differentiation efficiency of trisomic and disomic cell lines. To account for variability and epigenetic effects, we used isogenic iPSC lines as well as iPSCs from different individuals. The cells were differentiated using a monolayer culture, CHIR99021 induction protocol. Differentiation efficiency was assessed by measuring the amount of CD34^+^/CD31+ cells *via* flow cytometry. We found no significant differences between the percentages of endothelial cells generated at day 5 of trisomic (DSV-iPSC) *vs.* isogenic (isoDSV-iPSC) and disomic (SR2-iPSC) differentiation. With some variability, all cell lines had an endothelial differentiation efficiency of about 15%. Figure 4A shows the differentiation results for DSV-iPSCs and SR2-iPSCs.

#### Proliferation assays

To investigate how genome-wide expression dysregulation effects endothelial cell function, the differentiated iECs were subjected to functional assays that target vasculogenic potential. The first assay measured endothelial proliferative sensitivity to different VEGF concentrations: 0.5 ng/mL, 2 ng/mL, and 20 ng/mL. These values were chosen on the basis of free VEGF secretions (ranging from 0.3 ng/mL to 17.5 ng/mL) in ascites, pleural effusions, plasma, and serum of patients diagnosed with various cancer types [59]. By comparing the effects of such variable VEGF additions in DSV-iECs and control HUVECs (Human Umbilical Vein Endothelial Cells), we found that DSV-iECs were less proliferative and had a diminished response to VEGF. At a concentration of 0.5 ng/mL VEGF, there was a significant increase in HUVEC proliferation, while DSV-iECs had no significant response. Notably, when the concentration of VEGF was increased to 20 ng/mL, the difference in proliferative potential between HUVECs and DSV-iECs became less significant (Figure 4B).

As an additive confirmation, we performed an EdU Proliferation Assay using DSV-iECs and the SR2-iECs. The proliferation efficiency was assessed as the percentage of cells in the G0/G1 and S/G2 phases of the cell cycle. The results demonstrated that DSV-iECs, in comparison to SR2-iECs, had a smaller percentage of cells in the S/G2 phases for each treatment condition. Furthermore, similarly to HUVECs, SR2-iECs exhibited a significant proliferative response following 0.5 ng VEGF addition. DSV-iECs were more responsive after the addition of 20 ng VEGF (Figure 4C and 4D).

Having obtained these results, we also screened our RNA-Seq data for the expression levels of three VEGF receptors: FLT-1 (VEGFR1), KDR (VEGFR2), and FLT-4 (VEGFR3). The goal was to evaluate VEGF receptor expression levels in light of the VEGF response sensitivity of DSV-iECs. All three VEGF receptors are significantly up-regulated in DSV-iECs (Figure 4E). In contrast to this up-regulation, DSV-iECs still elicited a weaker VEGF response compared to control SR2-iECs. This inverse relationship is suggestive of a more widespread angiogenic dysregulation in DS.

#### Tube formation and spheroid assays

**Figure 5:**
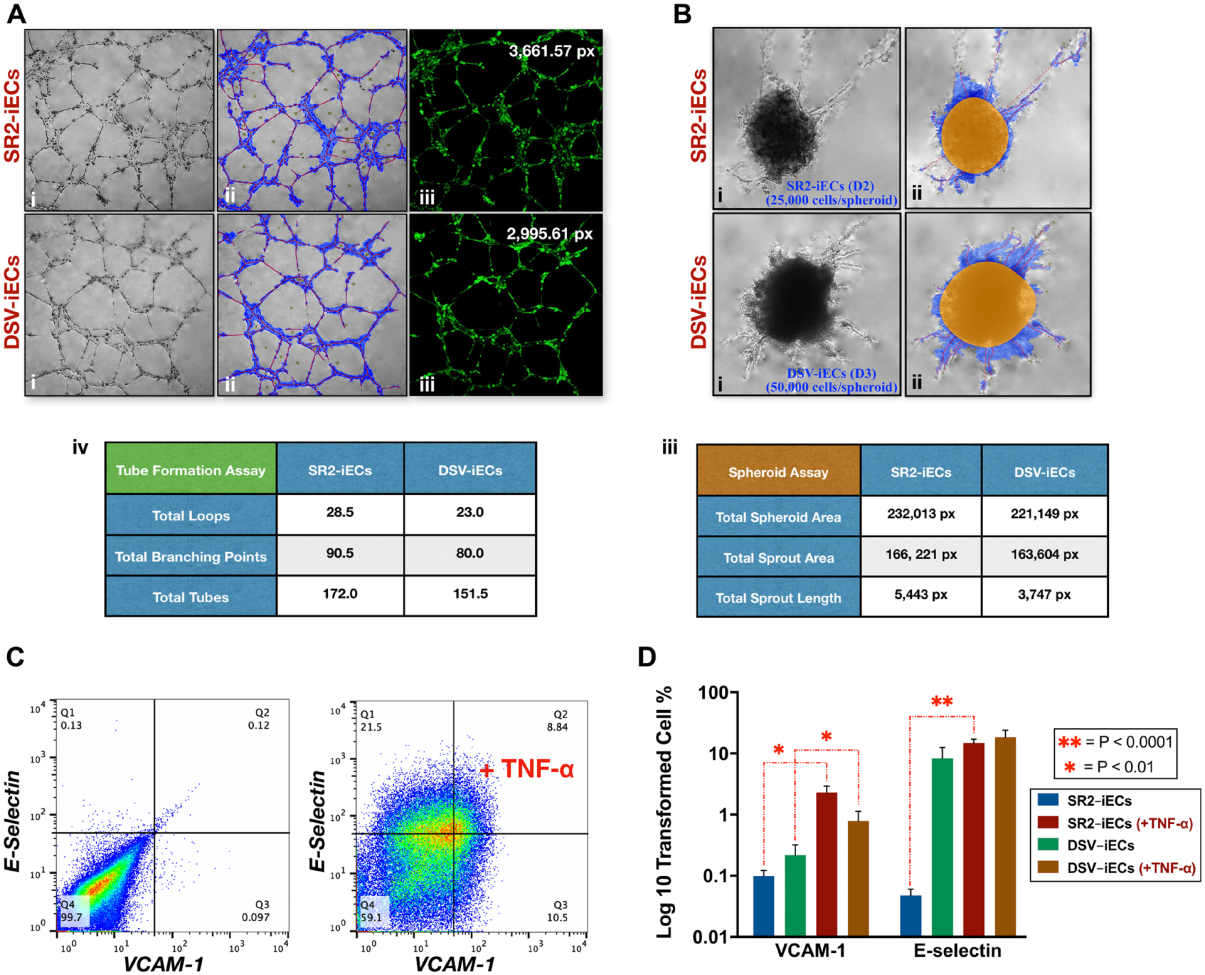
Tube formation, spheroid sprouting, and inflammatory response of disomic and trisomic iECs. (**A**) (i, ii) SR2-iEC and DSV-iEC phase contrast microscopy images of the Tube Formation Assay: prior to and following WimTube software analysis; (iii) SR2-iECs and DSV-iECs stained with cell-permeant dye Calcein-AM. The numerical values, reported in pixels (px), refer to total tube length; (iv) Tube Formation data table showing mean values for loops, branching points, and total number of tubes; (**B**) (i, ii) Phase contrast microscopy images of the Spheroid Assay: prior to and following WimSprout software analysis; (iii) Spheroid Assay data table showing mean values, which are reported in pixels (px), for total spheroid area, total sprout area, and total sprout length. (**C**) Representative image: flow cytometry results of iEC response to TNF-α stimulation. The sensitivity of the response was evaluated on the basis of VCAM-1 (CD106) and E-selectin (CD62E) cell surface expression. (**D**) Inflammatory Assay data incorporating four experimental replicates. Unlike the SR2-iEC line, DSV-iECs have VCAM-1 and E-selectin moderately expressed prior to TNF-α activation. Following the addition of TNF-α, DSV-iECs do not show as significant a difference in VCAM-1 and E-selectin expression like SR2-iECs. All statistical data in the Figure is presented as mean ± SEM.

We further evaluated the vasculogenic potential of DSV-iECs and control SR2-iECs *via* the Tube Formation Assay. We observed that DSV-iEC tubular extensions exhibited a thinner density, covered a smaller area of the culture wells, and had fewer branching points, loops, and total number of tubes in comparison to SR2-iECs (Figure 5A). DSV-iEC networks were also characterized by decreased stability and integrity. During incubation at 37°C and 5% CO_2_, following the 6 hr mark, DSV-iECs detached and degraded in a shorter time period *vs.* SR2-iECs (data not shown).

These results correlate with the Spheroid Assay data. SR2 endothelial spheroids demonstrated greater migratory potential: more sprouts, “detached” cells, and “edging” cells were noticeable in the culture well [60]. DSV-iEC spheroids, on the other hand, required an additional 24 hrs and twice the number of cells in order for spheroid area, sprout area, and cumulative sprout length to resemble control SR2-iECs (Figure 5B). Like the proliferation assays, these results also indicate that DSV-iEC angiogenic potential is lower than that of the disomic control.

#### Inflammation assay

To investigate the relationship between endothelial dysregulation and immune response, we conducted the Tumor Necrosis Factor-α (TNF-α) Inflammation Assay. TNF-α initiates the endothelial inflammatory cascade by activating transcription of selectins, cadherins, integrins, and CAM proteins. We decided to focus on E-selectin and VCAM-1 adhesion proteins because they play a key role in vascular transmigration [61, 62], and both proteins have also been implicated in tumor cell recruitment during inflammation [62, 63]. According to our flow cytometry data, following TNF-α stimulation, DSV-iECs showed a decreased activation of surface proteins VCAM-1 (CD106) and E-selectin (CD62E) compared to control SR2-iECs (Figure 5C and 5D). Such a diminished response possibly reflects the already present up-regulation of VCAM-1 and E-selectin prior to TNF-α stimulation. This could be indicative of endothelial dysfunction and/or other mediators influencing VCAM-1 and E-selectin expression.

## DISCUSSION

The Down syndrome phenotype is characterized by angiogenic, ECM-associated, and immune response imbalances [18, 64]. All of these factors, which rely on endothelial functionality, are key agents that tumors employ to create a favorable niche for growth, and ultimately, metastasis. Previous studies have shown that T21 has the potential to induce endothelial dysfunction as early as the progenitor stage, but the extent of this biological impact varies between DS individuals [65, 66]. Our data shows no significant difference between trisomic and disomic iPSC endothelial differentiation efficiency, yet functional assays show that impairment is evident in trisomic endothelial cells. These results acknowledge the possibility that T21 alters endothelial functionality throughout endothelial maturation-highlighting the consideration that T21 potentially utilizes a combination of gene dosage and microenvironmental/biochemical cues to elicit a temporally progressive endothelial impairment.

This mechanistic flexibility may form the framework as to why DS patients are prone to exhibiting a leukemic phenotype, yet are more resistant to solid tumor development and metastasis. In our Chromosome 21 cancer-related gene panel, all leukemia-associated genes were up-regulated, and the majority of solid tumor-associated genes were down-regulated. Our genome-wide bioinformatic analysis aligns with this expression trend. Trisomic endothelial cells showed a greater predisposition toward up-regulating leukemia-associated oncogenes and down-regulating solid tumor-associated oncogenes with a potential inclination toward tumor suppressor up-regulation. These results present the possibility that T21, on a genome-wide level, is challenging solid tumor development in a dualistic fashion: pairing down-regulation of oncogenes with up-regulation of tumor suppressors.

To further evaluate T21 impact on solid tumor development, bioinformatic pathway analysis was performed to gain insight into how T21 gene expression alterations may effect the endothelial microenvironment, which is a crucial component of the tumor niche. The more dynamic the tumor-niche interactions become, the greater the likelihood of metastasis-inducing signaling mechanisms. These mechanisms can lead to an increase in tumor proliferation, ECM anchorage, and immune evasion [67]. In consideration of this factor, statistical significance (*p*-value, FC) of gene expression, and pathway interconnectivity, we evaluated signaling pathways that regulate proliferation, cytoskeletal rearrangement, ECM remodeling, and inflammation. By thoroughly studying these pathways (HIF-1, EDN1/EDNRB, F3, CCL25/CCR9, actin-cytoskeleton, CCL2, IL-33, IL-1, EGFR), we firstly observed that Chromosome 21-specific gene expression levels did not exhibit as significant a FC in expression compared to genes mapped along other chromosomes (Chr. 1, Chr. 2, Chr. 4, Chr. 5, Chr. 6, Chr. 7, Chr. 9, Chr. 11, Chr. 17, Chr. 18, Chr. 19). Additionally, 14 out of the 15 selected, most significantly expressed, non-Chromosome 21 genes were down-regulated in DSV-iECs compared to disomic iECs. These two aspects introduce the possibility that T21 may regulate the endothelial microenvironment to a greater extent *via* genome-wide alterations *vs.* Chromosome 21-specific gene dosage effects.

Evaluation of the endothelial microenvironment from a proliferative perspective entails a focus on angiogenic factors, which mediate crosstalk between the tumor niche and endothelial cells. To promote tumor vasculature support, the homeostatic balance of pro- and anti-angiogenic signaling must shift in favor of pro-angiogenic overexpression. This aspect underlies the current interest in identifying key angiogenic players (ex., PDGF, SCF, ILs, TGF-β) that could serve as therapeutic targets capable of shutting off the tumor’s angiogenic “on” switch [68]. In DS, the presence of a possible anti-angiogenic microenvironment leading to the suppression of solid tumor growth was suggested in a study of a DS mouse model, which showed DSCR1 suppression of VEGF signaling [69].

Our DSV-iEC model revealed EDN1, ITGB3, and F3 genes as most significantly expressed in angiogenic/proliferative pathways. Additionally, the positive feedback loops that EDN1, ITGB3, and F3 share with inflammatory genes, such as IL-8, CXCL-1 (GRO-1), IL-33, CCL2, IL-6, and IL-1β, further emphasizes the significant role of this select gene set in angiogenesis. EDN1, ITGB3, and F3 have been implicated in tumor progression [70–72], but they have not been well studied with respect to DS. Their down-regulated expression, as shown in our DSV-iECs, also supports the potential presence of an anti-angiogenic microenvironment that may prevent solid tumor growth. Furthermore, this gene expression data, coupled with bioinformatic pathway analysis, correlates with our proliferation functional assays. DSV-iECs exhibited a reduced proliferative rate following VEGF addition, despite the increased gene expression levels of three VEGF receptors. DSV-iECs also spent significantly more time in the G0/G1 phases of the cell cycle in comparison to disomic iECs.

By pairing proliferative potential with migratory capability in the endothelial microenvironment, the pre-metastatic tumor transitions toward a more invasive phenotype. The extent of invasiveness is impacted by the distance between primary and secondary tumor sites and disruption in ECM composition, which has been linked to metastatic onset. During cancer progression, overexpression of MMPs—involved in degrading collagen, laminin, fibronectin, and proteoglycans—can induce porosity in the ECM basement membrane. This enables cancer cells to more easily bypass the ECM and achieve intravasation [73, 74]. Cancer cell protrusion of the ECM is also accompanied by dynamic actin polymerization and rearrangements. The cytoskeletal protrusions adhere to the ECM, and actin contractile machinery promotes cancer cell movement into the ECM membrane [75].

In consideration of this interplay between ECM composition and actin contractility, our DSV-iEC model exhibits down-regulated expression of MMP-1 and MMP-10 genes, as well as an up-regulation of HAPLN1, which plays a key role in creating proteoglycan-hyaluronic acid (HA) aggregates. This HA-based matrix imparts further stiffness onto the ECM membrane, which is associated with decreased migration [76]. Additionally, trisomic iEC down-regulated expression of the ACTG2 gene, which is part of the actin cytoskeletal complex, may be indicative of limited actin contractility and cell motility. Our functional assays support this possibility. The low integrity, density, and number of DSV-iEC tube formations as well as decreased spheroid area and sprout length offer support for a potentially reduced ECM-cytoskeletal dynamic in DS, which does not reflect the elevated migratory interactions typically associated with metastasis.

In addition to proliferative and migratory capabilities, the tumor niche also utilizes the endothelial microenvironment to evade immune detection. Individuals diagnosed with DS often have an impaired immune system [77], and it was hypothesized that T21-induced interferon signaling results in such chronic immune dysregulation [78]. In addition to this, DS adults also exhibit increased cytokine production (TNF-α, IFNγ, etc.) from peripheral blood mononuclear cells (PBMCs), which are highly involved in inflammatory processes [79]. Previous cancer research has shown that such inflammatory conditions are linked with tumor onset, and hypoxia plays a key role in this regard. Hypoxic conditions encourage oxidative damage, mutations, and the survival of more resistant, “stem-like” tumor cells that will undergo metastasis. Furthermore, the more delayed the inflammatory response, the greater is the opportunity for the tumor niche to release cytokines, chemokines, and exosomes for the purpose of priming secondary metastatic tissue sites [80].

Relative to hypoxia, our inflammatory genes of interest (CCL2, IL-8, IL-6, IL-1β, SERPINB2 [PAI2], IL-33, CXCL-1 [GRO-1], and APOE) are downstream of NF-κB, which activates transcription of HIF-1. In DSV-iECs, all of these genes are down-regulated with the exception of APOE. Our TNF-α inflammatory response functional assay aligns with this result: compared to disomic iECs, DSV-iECs were less responsive following TNF-α stimulus. Another interesting aspect to note is that DSV-iECs had elevated VCAM-1 and E-selectin expression prior to TNF-α addition. This factor raises the possibility that DSV-iEC inflammatory response may be impacted by endothelial dysregulation and/or other mediators, such as already up-regulated cytokine levels [79]. Furthermore, APOE’s activating and inhibiting inflammatory activity [81], combined with the down-regulation of mentioned inflammatory response genes, is an additional point to consider as this may potentially contribute toward a prolonged state of inflammation.

Taking into account all of these aspects, this work identified the following factors that may offer insight into the question of why DS individuals exhibit an elevated leukemic precedence and decreased solid tumor growth: (1) decreased proliferative and migratory capability; (2) a potentially prolonged inflammatory state; (3) down-regulation of genome-wide solid tumor-associated oncogenes; and (4) an up-regulation of genome-wide leukemia-associated oncogenes. These factors also highlight the widespread involvement of the tumor niche during pre-metastatic phases of cancer development and the importance of evaluating the endothelial microenvironment from a variety of molecular perspectives. The use of iPSCs and directed differentiation protocols provide a new and powerful tool to continue gaining more insight into the biology of DS, endothelial development, the solid tumor niche, and a wide array of human diseases. As more differentiation models become available, such as hematopoietic stem cells, the types of experiments that can be done and their correct interpretation will grow.

## MATERIALS AND METHODS

### Cell culture

Skin fibroblasts (FB1, FB2) were obtained from patients (Caucasian females aged 2- and 3-y-old) at Ann & Robert H. Lurie Children’s Hospital. The fibroblasts were cultured in DMEM medium (Life Technologies) supplemented with 10% FBS (Fisher Scientific), NEAA (Fisher Scientific), and HEPES (Fisher Scientific). Trisomic (DSV) and disomic (SR2) iPSCs were derived *via* over-expression of Sox2, c-Myc, Oct4, and Nanog in commercially purchased fibroblasts. Additional information is provided in Galat *et al*. (2016, 2017). Prior to differentiation, the cells were maintained on Matrigel-coated culture dishes in mTeSR1 medium (STEMCELL Technologies). The isoDSV-iPSCs were obtained *via* the spontaneous loss of an extra copy of Chromosome 21 in DSV-iPSCs. H9-ESCs (WA09) were purchased from WiCell. HUVECs were kindly provided by the Hendrix laboratory, which purchased the cells from ATCC (PCS-100-013).

### Endothelial differentiation

Endothelial differentiation of DSV-iPSCs, isoDSV-iPSCs, H9-ESCs, and SR2-iPSCs was induced *via* the addition of CHIR99021 (STEMCELL Technologies) and vascular endothelial growth factor (VEGF_165_) (R&D Systems) to the culture medium. On day 4, the differentiated iECs were isolated by immuno-selection of CD31^+^CD144^+^ cells *via* a magnetic column (Miltenyi Biotec). Following this, the iECs were grown on fibronectin-coated (10 μg/mL) (BD Biosciences) plates and cultured in VascuLife EnGS medium (LifeLine) at 37°C and 5% CO_2_.

### Flow cytometry analysis

To verify the endothelial marker expression the iECs were analyzed *via* flow cytometry. The cells were harvested with StemPro Accutase (ThermoFisher Scientific), washed with ice-cold FACS buffer (PBS + 1% FBS + 2 mM EDTA), and incubated with conjugated antibodies CD31 PE, CD34 FITC, VE-Cadherin APC (Miltenyi Biotech) for 30 minutes at 4°C. Following this, the cells were washed with a 0.5% BSA/PBS solution. Data collection was performed via the FACSCalibur (BD Biosciences) and analyzed with the FlowJo software (version 10.5.3).

### Immunocytochemistry

The following procedures were all performed at room temperature. iECs were fixed with 3.2% paraformaldehyde for 30 min and permeabilized for 5 min with 0.1% Triton-x-100 in PBS. The cells were then treated with Dako Protein Block for 25 min in order to prevent nonspecific antibody binding. Following this, iECs were incubated with the following mouse anti-human, primary antibodies: VE-Cadherin (BD Biosciences) (1 hr) and VWF (R&D Systems) (3 hrs). After washing the cells 3× with Dako Washing Buffer (WB), the appropriate Alexa Fluor-conjugated secondary antibodies (Invitrogen) were added to cell culture wells; the incubation time was 45 minutes. All antibody dilutions were performed according to manufacturers’ instructions. Samples were then washed once more with WB and incubated with DAPI (Sigma Aldrich) for 3 minutes. The immunofluorescent cells were visualized with Leica DM IRB inverted microscope system (Leica, Germany) equipped with a digital camera Retiga 4000R (Qlmaging, Canada), which was controlled with Openlab software version 5.0.2 (Perkin-Elmer).

### Proliferation assays

Assay #1: 30,000 control and trisomic cells were seeded per well onto fibronectin-coated (10 μg/mL) (BD Biosciences) 6-well plates. The cells were cultured in VascuLife EnGS medium (LifeLine) containing varying VEGF concentrations (0.5 ng/mL, 2 ng/mL, and 20 ng/mL). When one of the cell lines reached confluence, all cells for a particular VEGF concentration were harvested with StemPro Accutase (ThermoFisher Scientific) and a cell count was performed.

Assay #2: On day 6, utilizing the Click-iT EdU Flow Cytometry Assay Kit (cat #: C10425) and following the manufacturer’s protocol, the cells were harvested, labeled, and analyzed via the Accuri flow cytometer. Endothelial proliferation potential was assessed relative to cytometric DNA synthesis measurement (G0/G1 and S/G2 cell cycle phases).

### Tube formation assay

Matrigel (Corning) was thawed overnight at 4°C. The following morning, matrix coating was added to 12-well cell culture plates, which were incubated for 30 min at 37°C and 5% CO_2_. iECs were seeded at a density of 2.75 × 10^5^ cells per well and incubated for 6 hrs in VascuLife EnGS medium (LifeLine). After the incubation period, the cells were treated with the cell permeable dye Calcein-AM (2 μg/mL) and incubated for 30 min at 37°C and 5% CO_2_. Afterwards, the 12-well cell culture plates were ready for tube network visualization under the Leica DM IRB inverted microscope system (Leica, Germany) equipped with a digital camera Retiga 4000R (Qlmaging, Canada).

### Spheroid assay

iECs were cultured in VascuLife EnGS complete medium (LifeLine) on 6 cm dishes coated with fibronectin (10 μg/mL) (BD Biosciences). Prior to harvesting the cells, methocel solution was prepared by mixing methylcellulose powder (4,000 cP) (Sigma-Aldrich) with preheated (60°C) VascuLife EnGS basal medium (LifeLine). Following this, an equivalent amount of Vasculife basal medium containing 5% FBS was added to the mixture and the solution was stirred overnight at 4°C. The solution was stored at 4°C until cell monolayers were grown. The confluent cells were harvested with StemPro Accutase (ThermoFisher Scientific), centrifuged, and resuspended in 20% methocel + 80% VascuLife complete medium. Following this, 30 uL cell suspensions were used to generate 3D spheroids according to the JoVE hanging drop protocol. Next, a collagen-neutral solution was prepared by mixing collagen (Type I) (ThermoFisher Scientific) + 10× EBSS (ThermoFisher Scientific) + 0.1 N NaOH + 0.1 N HCl. This mixture was combined in a 1:1 ratio with Vasculife, containing 20% FBS, 0.5% Methocel, and 100 ng/mL VEGF. Afterward, the spheroids were plated in the following manner: the first layer of the combined mixture was added to a 4-well culture plate, followed by the spheroid, and then another layer of the mixture. The spheroids were cultured at 37°C and 5% CO_2_ for 3 days and imaged via a light microscopy Nikon D100 digital SLR camera (Tokyo, Japan) on a Leica DM IRB inverted microscope.

### Inflammatory response assay

Confluent iEC monolayers were incubated with TNF-α (10 ng/mL) (Biolegend) for 6 hrs at 37°C and 5% CO_2_. Following TNF-α treatment, the cells were harvested with StemPro Accutase (ThermoFisher Scientific), washed with ice-cold FACS buffer (PBS + 1% FBS + 2 mM EDTA), and incubated with E-selectin APC (Miltenyi Biotech) and VCAM-1 FITC (eBioscience) conjugated antibodies for 30 min at 4°C. Following this, the cells were washed with a 0.5% BSA/PBS solution. EC inflammatory response to TNF-α was measured *via* flow cytometry in light of VCAM-1 and E-selectin surface expression percentage. Data collection was performed via the FACSCalibur cytometer (BD Biosciences) and analyzed via the FlowJo software (version 10.5.3).

### RNA isolation

Total RNA was extracted with the RNeasy Mini Kit (Qiagen) via the instructions provided in the manufacturer’s protocol. RNA quality and concentration were assessed via the Nanodrop.

### Microarray analysis

RNA aliquotes were submitted to University of Chicago Genomics Facility. The RNA samples were reverse transcribed into cDNA, which was hybridized onto a HumanHT-12 v4BeadChip that was scanned by Illumina iScan. The acquired data was processed and normalized *via* the iScan Control software. Gene expression comparisons were obtained using the R Studio software (Bioconductor package).

### RNA sequencing analysis

Aliquots of RNA were submitted to Northwestern University’s NUSeq Core. The mRNA library was prepared and the samples were analyzed using HiSeq 4000 Sequencing 50bp, Single Reads. The obtained list of differentially expressed genes was further analyzed using MetaCore (Clarivate Analytics version 19.4/build 69900) and R Studio software (version 3.6.1). The gplots, pheatmap, and enhancedvolcano packages were incorporated into the R script in order to generate the heatmap and volcano plot.

### Real time qPCR

The High-Capacity RNA-to-cDNA kit (Applied Biosystems) was used to reverse transcribe the isolated RNA. Each reaction tube included up to 2 ug of RNA. The reverse transcription reaction was performed according to manufacturer instructions via the MBS Satellite (0.2 G) Thermal Cycler (ThermoFisher Scientific). The qPCR reaction mix was prepared by adding 12 ng of cDNA from each sample to the PowerUp SYBR Green Master Mix (2×) (Applied Biosystems). qPCR was performed (Standard Cycling Mode, primer T_m_ < 60°C) *via* the 7500 Fast Real-Time PCR system (Applied Biosystems). The 7500 v2.3 software was used for data collection and gene expression comparisons (2^-ΔΔCT^ method). Primer sequences provided in Supplementary Table 1.

## SUPPLEMENTARY MATERIALS



## Abbreviations

ALL: acute lymphoblastic leukemia
AMKL: acute megakaryocytic leukemia
DS: Down syndrome
DSCR: Down syndrome critical region
CNAs: copy number alterations
iECs: iPS-produced endothelial cells
ECM: extracellular matrix
EDN1: Endothelin-1
ESCs: embryonic stem cells
F3: Tissue factor
FC: fold change
HA: hyaluronic acid
HIF-1: Hypoxia Inducible Factor-1
iPSCs: induced pluripotent stem cells
HUVECs: human umbilical vein endothelial cells
ITGB3: Integrin beta-3
MMP: matrix metallopeptidase
PBMCs: peripheral blood mononuclear cells
T21: trisomy of Chromosome 21
TCGA: The Cancer Genome Atlas
TME: tumor microenvironment
TNF-α: tumor necrosis factor-alpha
VWF: von Willebrand factor
