# Supplementary material for: Down syndrome iPSC model: endothelial perspective on tumor development
Source: Oncotarget. 2020 Sep 8;11(36):3387–404. doi: 10.18632/oncotarget.27712 (PMC7486695; doi:10.18632/oncotarget.27712)
Supplement: Supplementary file 1 [file oncotarget-11-3387-s001.pdf]

Down syndrome iPSC model: endothelial perspective on tumor development

SUPPLEMENTARY MATERIALS

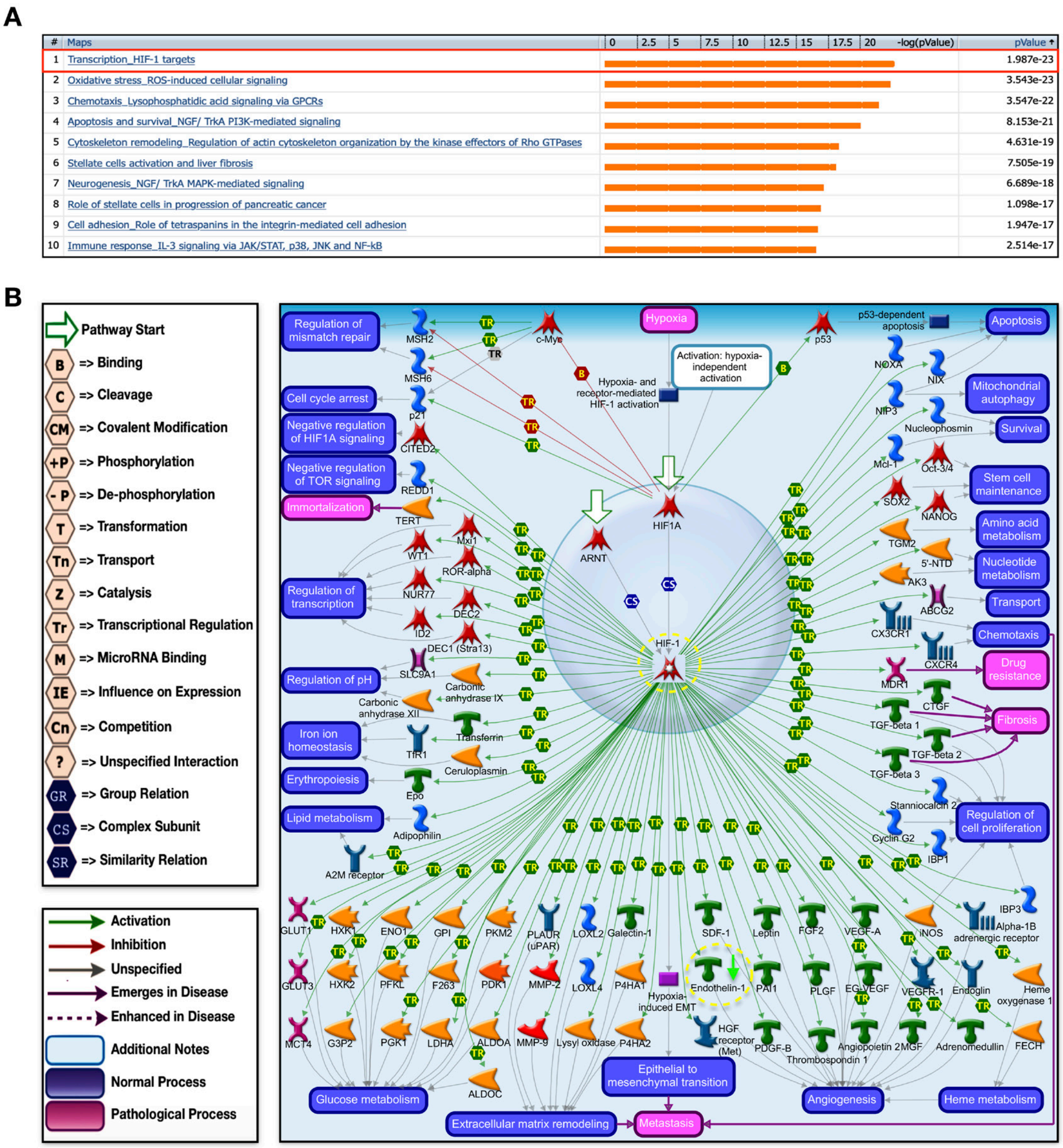

#	Maps	-log(pValue)	pValue
1	Transcription_HIF-1 targets		1.987e-23
2	Oxidative stress_ROS-induced cellular signaling		3.543e-23
3	Chemotaxis_Lysophosphatidic acid signaling via GPCRs		3.547e-22
4	Apoptosis and survival_NGF/ TrkA PI3K-mediated signaling		8.153e-21
5	Cytoskeleton remodeling_Regulation of actin cytoskeleton organization by the kinase effectors of Rho GTPases		4.631e-19
6	Stellate cells activation and liver fibrosis		7.505e-19
7	Neurogenesis_NGF/ TrkA MAPK-mediated signaling		6.689e-18
8	Role of stellate cells in progression of pancreatic cancer		1.098e-17
9	Cell adhesion_Role of tetraspanins in the integrin-mediated cell adhesion		1.947e-17
10	Immune response_IL-3 signaling via JAK/STAT, p38, JNK and NF-kB		2.514e-17

Supplementary Figure 1: HIF-1 pathway. (**A**) On the basis of gene expression values and molecular interactions, the MetaCore software calculated and attributed the lowest *p*-value to the HIF-1 pathway (Map ID: 448). (**B**) The HIF-1 pathway accompanied with a guide modeled after "Metacore's Quick Reference Guide." The HIF-1 complex and the Endothelin-1 (EDN1) gene are highlighted, and the green arrow symbolizes down-regulated gene expression.

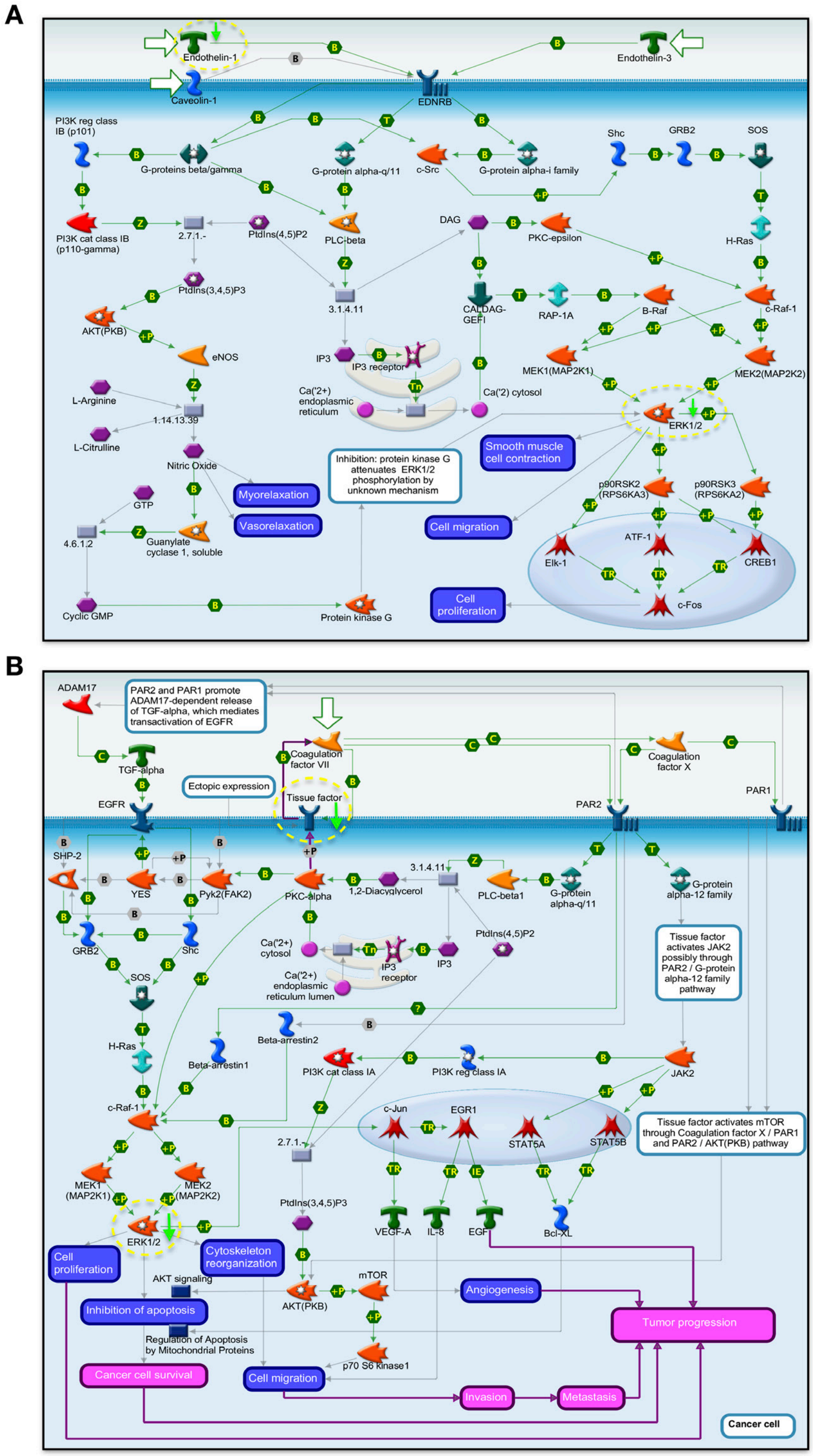

Supplementary Figure 2: EDN1/EDNRB and tissue factor pathways. (**A**) MetaCore pathway showing EDN1/EDNRB signaling (Map ID: 2273), which is upstream of the ERK1/2 complex. EDN1 and ERK1/2 down-regulation is shown *via* the green arrows. (**B**) Tissue factor (F3) signaling pathway (Map ID: 5015), which shows Tissue factor's upstream regulation of ERK1/2. The green arrows indicate down-regulated gene expression.

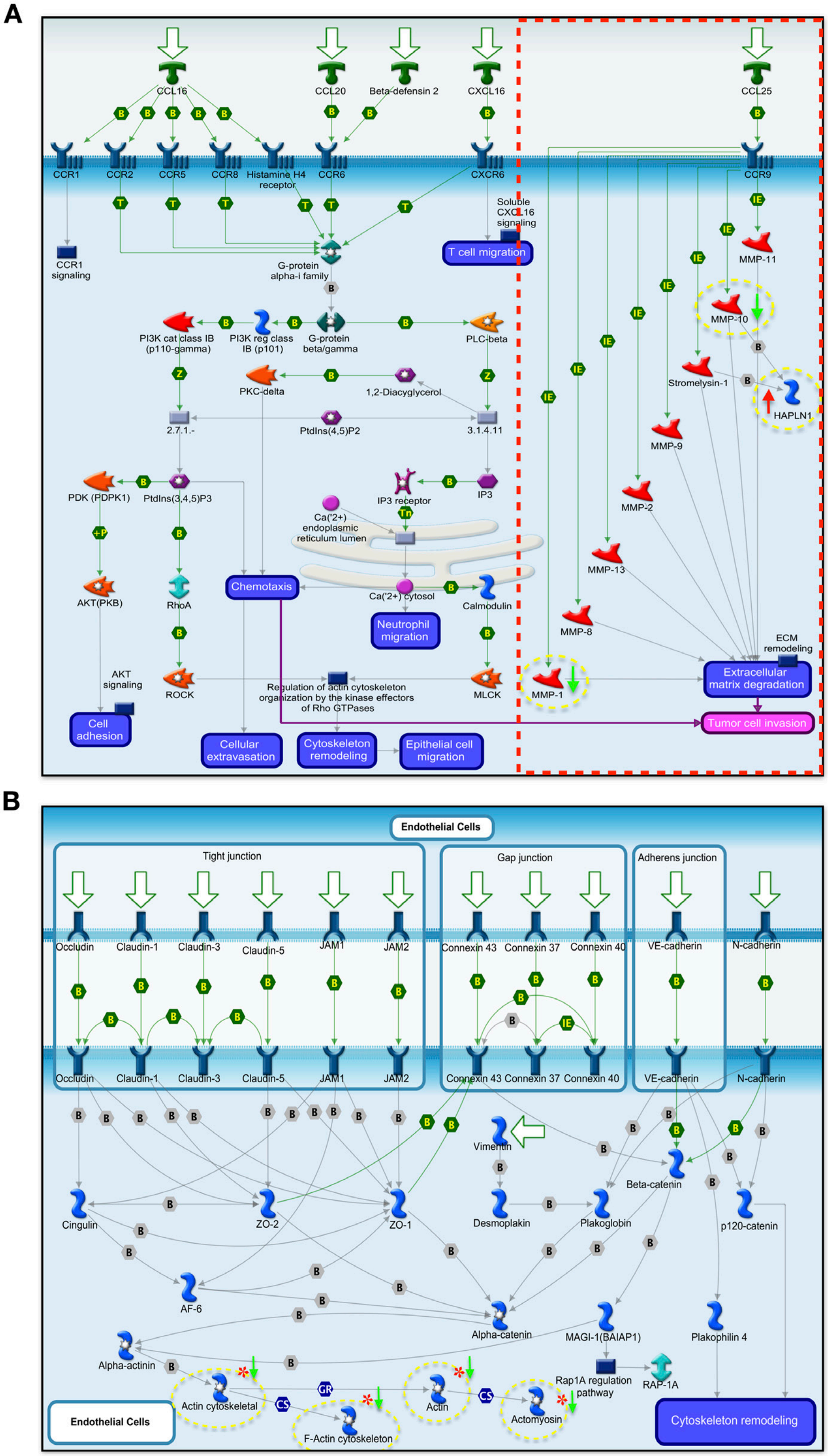

Supplementary Figure 3: ECM (CCL25) and cytoskeletal remodeling pathways. (**A**) Cell migration pathway showing the interconnectivity of CCL16, CCL20, Beta-defensin 2, CXCL16, and CCL25 signaling (Map ID: 6963). The CCL25/CCR9 pathway, which has a direct link to ECM remodeling, has been denoted in red. MMP-1, MMP-10, and HAPLN1 genes are highlighted, and gene expression levels are indicated by red [up-regulation] and green [down-regulation] arrows. The HAPLN1 gene was incorporated into the pathway *via* MetaCore's Pathway Map Creator application. (**B**) MetaCore pathway depicting cell adhesion and cytoskeletal remodeling (Map ID: 745). The actin complexes are highlighted in yellow. The red stars indicate the presence of the ACTG2 gene in each of these complexes. The green arrows refer to the down-regulated ACTG2 expression.

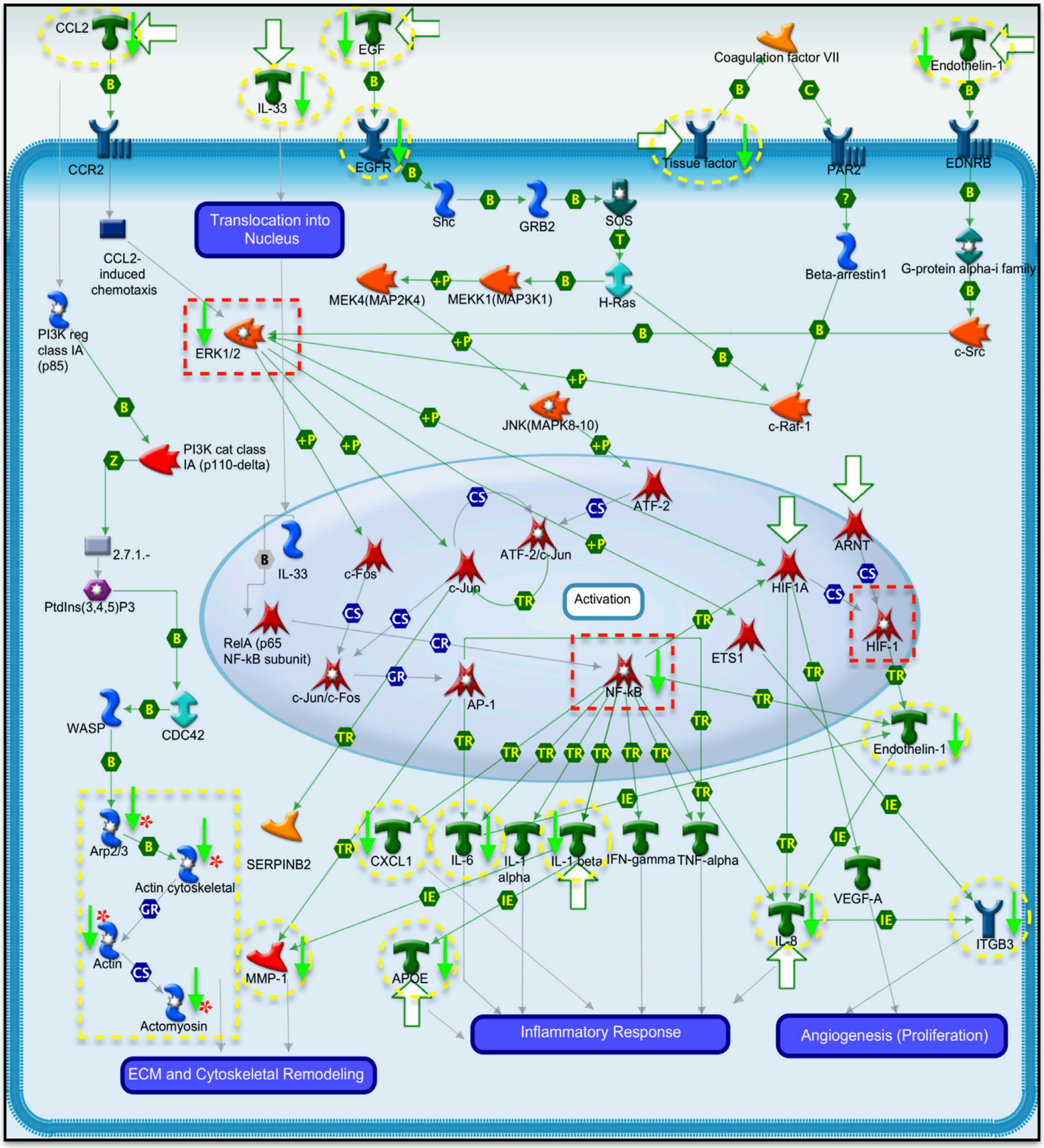

Supplementary Figure 4: Proliferation and inflammation pathways. A combined proliferation and inflammation pathway was created utilizing the Pathway Map Creator application, which incorporates data from published MetaCore pathways. Within this newly created pathway, the interconnectivity with regard to ERK1/2 and NF-κB complexes is more clearly seen. Furthermore, this pathway map demonstrates how proliferation and inflammation pathways are in constant communication and impact ECM remodeling and cytoskeletal rearrangements. The green arrows symbolize down-regulated gene expression.

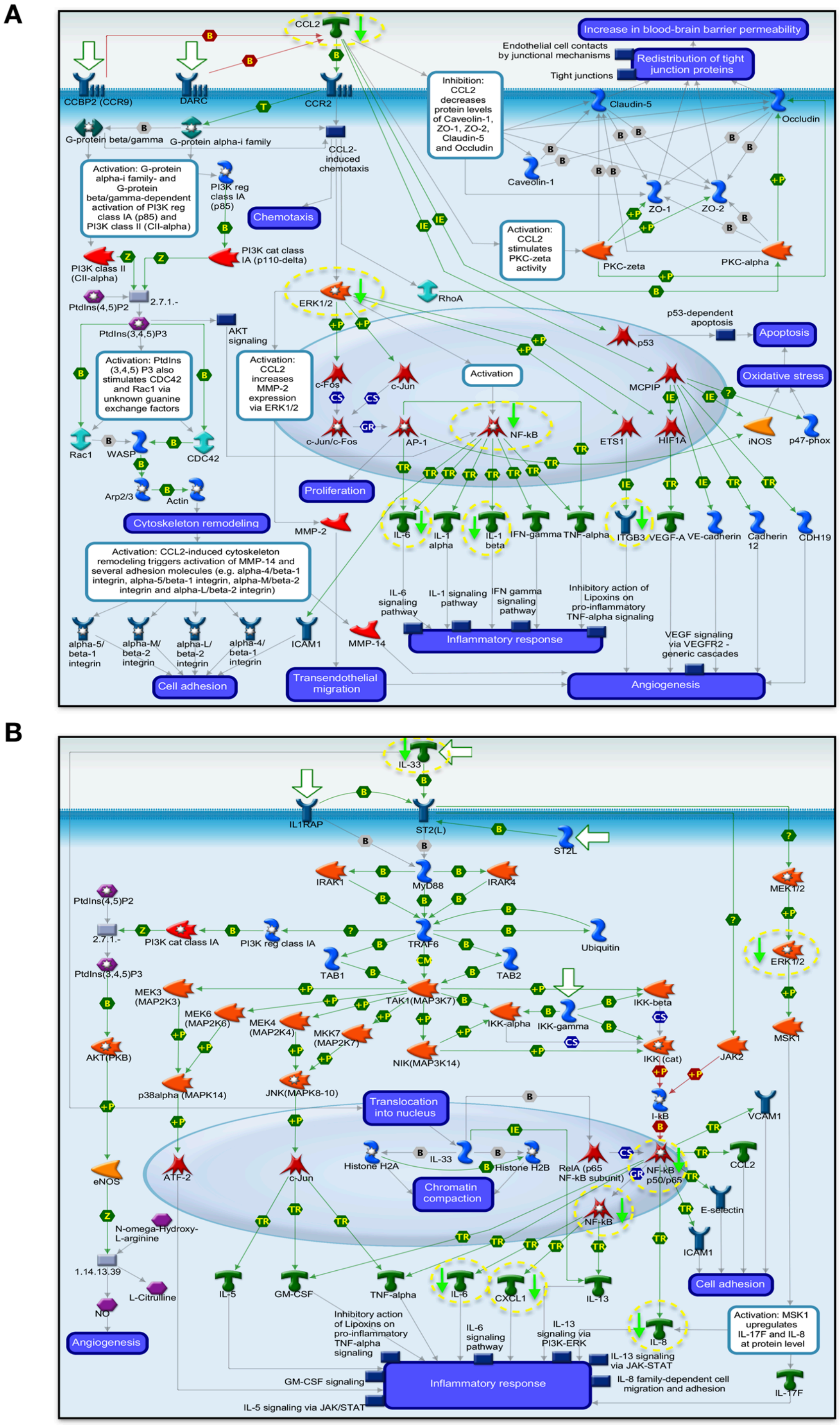

Supplementary Figure 5: CCL2 and IL-33 pathways. (**A**) MetaCore CCL2 pathway (Map ID: 6515) showing CCL2 as an upstream regulator of the ERK1/2 complex (effects ITGB3 expression) and the NF-κB complex (effects IL-6 and IL-1β expression). (**B**) IL-33 pathway (Map ID: 6684), another regulator of ERK1/2 and NF-κB, impacts IL-6, CXCL1, and IL-8 expression levels. Down-regulated gene expression is shown *via* green arrows.

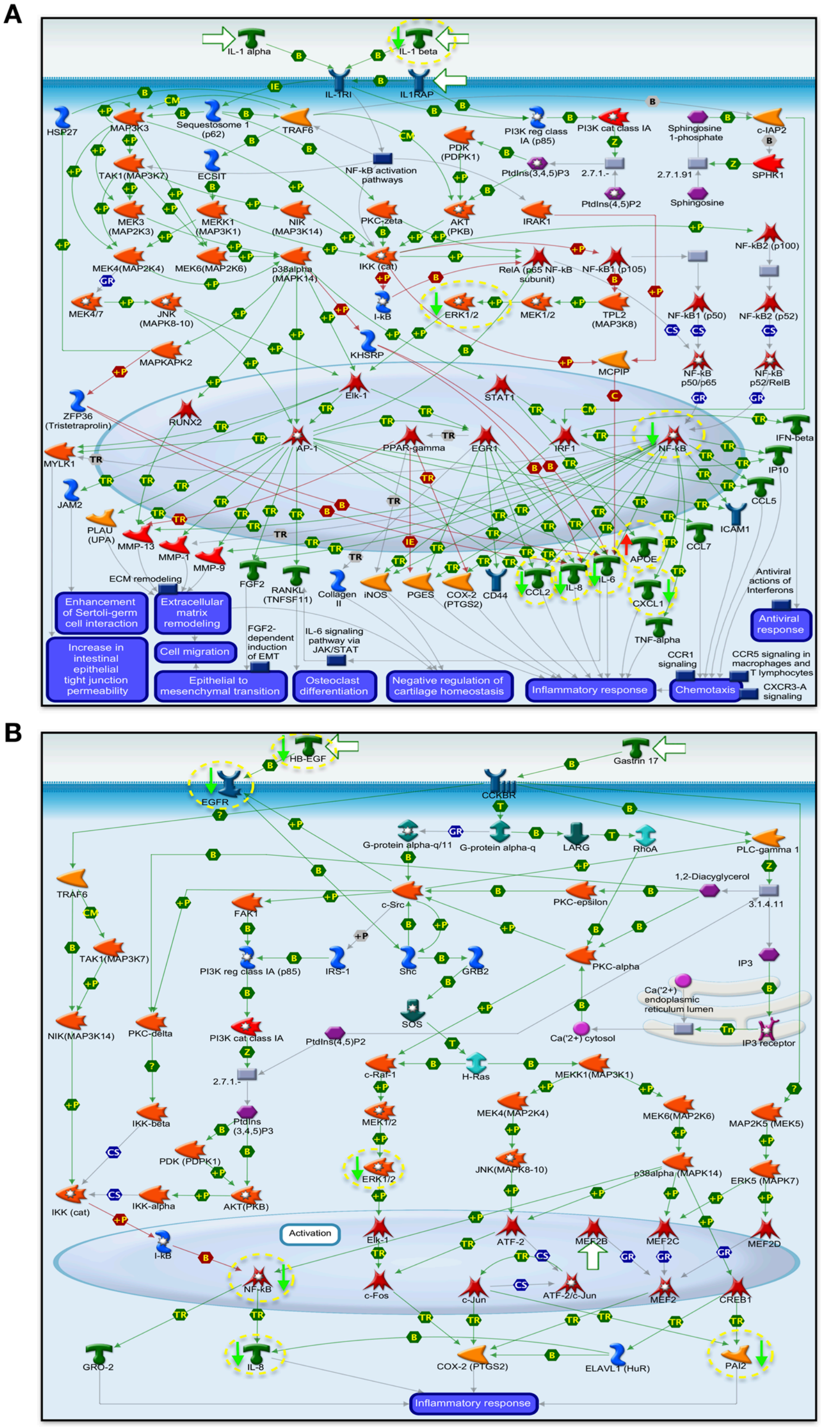

Supplementary Figure 6: IL-1 and EGFR pathways. (**A**) IL-1 pathway (Map ID: 658) showing ERK1/2 and NF-κB as downstream targets. NF-κB is a key regulator of APOE, CXCL1, IL-8, CCL2, and IL-6 genes. The APOE gene was incorporated into the pathway *via* the Pathway Map Creator application. (**B**) EGFR pathway (Map ID: 3136) showing downstream regulation of ERK1/2 and NF-κB signaling. The genes most significantly impacted are SERPINB2 (PAI2) and IL-8. Gene expression levels are indicated by red [up-regulation] and green [down-regulation] arrows.

Supplementary Table 1: Endothelial gene sequences

GENE	FORWARD PRIMER	REVERSE PRIMER
GADPH	GGCTGAGAACGGGAAGCTTGTCAT	CAGCCTTCTCCATGGTGGTGAAGA
APOE	CCAGCGGAGGTGAAGGAC	CGCTTCTGCAGGTCATCG
HAPLN1	GATACTGTTGTGGTAGCACTGG	TGCTGCGCCTCGTGAAAATTGAG
CCL2	CAGCCAGATGCAATCAATGCC	TCGAATCCTGAACCCACTTCT
